# A Comprehensive Review of the Development of Carbohydrate Macromolecules and Copper Oxide Nanocomposite Films in Food Nanopackaging

**DOI:** 10.1155/2022/7557825

**Published:** 2022-03-05

**Authors:** Mohammad Mesgari, Amir Hossein Aalami, Thozhukat Sathyapalan, Amirhossein Sahebkar

**Affiliations:** ^1^Department of Food Science and Technology, Faculty of Agriculture, Ferdowsi University of Mashhad, Mashhad, Iran; ^2^Department of Biology, Mashhad Branch, Islamic Azad University, Mashhad, Iran; ^3^Department of Academic Diabetes, Endocrinology and Metabolism, Hull York Medical School, University of Hull, Hull, UK; ^4^Biotechnology Research Center, Pharmaceutical Technology Institute, Mashhad University of Medical Sciences, Mashhad, Iran; ^5^Applied Biomedical Research Center, Mashhad University of Medical Sciences, Mashhad, Iran; ^6^Department of Medical Biotechnology and Nanotechnology, Faculty of Medicine, Mashhad University of Medical Sciences, Mashhad, Iran; ^7^Department of Biotechnology, School of Pharmacy, Mashhad University of Medical Sciences, Mashhad, Iran

## Abstract

*Background*. Food nanopackaging helps maintain food quality against physical, chemical, and storage instability factors. Copper oxide nanoparticles (CuONPs) can improve biopolymers' mechanical features and barrier properties. This will lead to antimicrobial and antioxidant activities in food packaging to extend the shelf life. *Scope and Approach*. Edible coatings based on carbohydrate biopolymers have improved the quality of packaging. Several studies have addressed the role of carbohydrate biopolymers and incorporated nanoparticles to enhance food packets' quality as active nanopackaging. Combined with nanoparticles, these biopolymers create film coatings with an excellent barrier property against transmissions of gases such as O_2_ and CO_2_. *Key Findings and Conclusions*. This review describes the CuO-biopolymer composites, including chitosan, agar, cellulose, carboxymethylcellulose, cellulose nanowhiskers, carrageenan, alginate, starch, and polylactic acid, as food packaging films. Here, we reviewed different fabrication techniques of CuO biocomposites and the impact of CuONPs on the physical, mechanical, barrier, thermal stability, antioxidant, and antimicrobial properties of carbohydrate-based films.

## 1. Introduction

Plastic is a hazardous material and very challenging to decompose, one of the world's fundamental problems. The use of plastic has a role in ordinary life, particularly food packing and different accessories [1, 2]. About 63% of the current plastic waste comes from packaging purposes, and it is calculated that less than 14% is recyclable [3]. Recently, there is a more significant interest in using sustainable and degradable packaging, as they are usually functional and more environmentally friendly [2]. Therefore, there is a growing requirement for bio-based natural materials to resolve the waste disposal problems to an ensured magnitude. In this regard, biopolymers, especially those from renewable nonchemical resources, have been considered replacements of nonbiodegradable plastic supplies [4, 5].

Biopolymers are natural, environmentally friendly, nontoxic, and a real option to decrease nonbiodegradable and nonrenewable materials in the packaging productions [6, 7]. Proteins, polysaccharides, and lipids are the most common biopolymers utilized in the packaging materials [8]. Active food packaging (AFP) with natural antimicrobial agents is a suitable prospect for extending food products' shelf life, including fruit, meat, fish, and bread packaging [9]. To achieve active nanopackaging, degradable films have been combined from functional additives such as antioxidants and antimicrobials agents that may be transferred from packaging to food products to extend their shelf life [6, 10, 11]. Antioxidant-containing films could primarily prevent the oxidation of fatty foods [12].

Nanotechnology-arisen products are now used in numerous fields of life and have shown their potential from industry to basic sciences and medicine to develop new tools, systems, and drugs [13, 14]. Metallic nanoparticles (MNPs) synthesized by multiple unique methods and properties, which help their exploitation in independent fields, such as nanodiagnostics [15, 16], nanomedicine [17–19], antibacterial [19–21], antioxidant [19, 20, 22, 23], luminescence [24, 25], photocatalytic [24, 26], painting, ceramic, glass production, and food packaging industry [27–34]. Nanoparticles such as titanium, silver, zinc, and copper have been applied worldwide to form nanocomposite packaging materials with antimicrobial and antioxidant activities [7, 35–37].

The metal oxide nanostructure, especially copper oxide nanoparticles (CuONPs) structures, is interested in its potential importance in the biomedical field [38]. The CuONPs can inhibit the growth of microorganisms such as bacteria, fungi, viruses, and algae [39]. The size of nanoparticles and the high surface zone of CuO permit them to interact closely with the cell membrane and thus have an excellent antimicrobial potential [40, 41]. Copper ions destroy them by receiving and donating electrons, revealing high redox potential and interrupting microbial cell components [42]. The antibacterial and antioxidant activities of CuONPs have been extensively studied in some fields, such as the improvement of polymer nanocomposites and food packaging materials [43, 44]. The principal purpose of this review is to discuss the role of CuONPs in the active food packaging system. The food packaging biomaterials combined with CuONPs are classified by the polymer type used to fabricate food.

## 2. Food Packaging

The principal purpose of food packaging is to maintain the quality and protection of food products from unpleasant odors, preserve flavor, and also in storage, transportation and to increase their shelf life by protecting from hazardous microorganisms and their toxins, chemical composites, sunlight, temperature, permeable volatile compounds, oxygen, and humidity [45,46]. Alterations in packaging lead to quality packing and consumer-friendly access in managing the shelf life and biodegradable packing [47]. In this regard, biopolymers can be used as active food packaging or for food coverage purposes as biodegradable materials that participate in the cycle of nature, reducing environmental effects and oil dependency [48].

## 3. Active Food Packaging

Active food packaging films have been developed in recent years to increase the shelf life and protect the quality of food goods. Antioxidant activity in these types of active food films has mainly been focused on limiting or delaying lipid and protein oxidation. Unlike typical food packaging materials, active food packaging methods have adequate barriers against H_2_O and O_2_, humidity absorbers, flavor, carbon dioxide absorbers or emitters, ethylene gas (such as ethylene absorbers/oxidizing agents), antioxidation materials, and antimicrobial agents [49–54]. Depending on the functional elements set in the biopolymers, the packaging can offer biological activities for the packaged foods, such as antioxidant and antimicrobial shields [55]. In this regard, some nanoparticles, such as copper oxide nanoparticles, have been exploited as active nanostructured food packaging materials with the association of some macromolecule biopolymers [56–61]. In the following sections, important biopolymers in food packaging, the barrier features of the food packaging process, and their mechanical properties are summarily explained with particular attention to CuONPs in food packaging, antimicrobial, and antioxidant permeability.

### 3.1. Carbohydrate Polymers

Biopolymers originated from different natural sources such as polysaccharides, protein, or aliphatic polyesters (Table 1). They have been touted as intriguing alternatives to traditional polymers since they are renewable, self-sufficient, cost-effective, environmentally friendly, and biodegradable [62]. The carbohydrate polymers in food packaging supplies can be divided into several subgroups.

#### 3.1.1. Agar

Agar is a linear polysaccharide consisting of *ß*-1, 3 D-galactose, and *a*-1, 4-linked 3,6-anhydrous-L-galactose units with partial side-chain substituents such as sulfate ester, methoxyl group, and pyruvate [63, 64]. Agar is a fiber carbohydrate extracted from a group of seaweeds of the class Rhodophyceae. It is one of the most suitable materials because it is abundant, thermoplastic, biocompatible, and biodegradable [62]. Today, agar is widely used as a food packaging material due to its excellent film-forming ability, thermoplasticity, environmental adaptability, biodegradation, and water resistance. On the other hand, agar-based films have disadvantages in food packing, such as low-temperature stability, restricted oxygen, and low water barrier qualities [65, 66].

#### 3.1.2. Starch

Starch is a natural polysaccharide that performs essential functions in food production as high-quality components or additives. Structurally, starch is made of two macromolecules: amylose and amylopectin. Amylose is a linear polysaccharide, poly(*α*-1,4-glucopyranosyl), whereas amylopectin is poly (*α*-1, 4-glucopyranosyl) with many *a*-1, 6-glucopyranosyl branches [67, 68]. Starch-based films are among the most valuable classes of bio-based packaging material in terms of performance, adjustability to products, processing, and price [69]. The edible films from cereal and roots of starches have widely been developed and examined for their features. However, the studies on films created from legume starches are strangely limited [69]. Starch is commonly employed as an adhesive, additive, or thickening in food packaging. The benefit of starch is that it acts as a medium oil barrier. However, it may be faulty in a humid environment due to hydrophilic functional groups in molecule formation. Its crystalline region can form a parclose against external gases, which amylose films have a powerful impact than amylopectin films [70]. The starch-based film applied as a packaging material is too brittle. Starch independently cannot form edible films that possess the expected mechanical properties unless combined with bio-ingredients such as a bio-food package [71].

#### 3.1.3. Cellulose

Cellulose is a natural polymer made from corncobs, which is environmentally friendly and biodegradable. Cellulose makes up roughly 40%–50% of the total content of natural fibers and alters the chemical features of fiber plants. Plants create around 75 billion tons of cellulose every year, resulting in fantastic cellulose biopolymers [72]. The usual cellulose formula is (C6H10O5)n, with cellulose as the significant component. Cellulose is rarely seen in nature in its purest form [73].

Moreover, colorants, gums, lipids, tannins, lignin, and hemicellulose are often mixed with cellulose. The 1,4-glycosidic bonds linking the cellulose and *ß*-glucopyranoside residues are utilized to reveal cellulose's prime shape [74, 75]. Because of their recyclability and degradability, cellulose nanocomposite films have gotten much attention. However, their high cost and susceptibility to water limit their use [70].

(1) Bacterial cellulose (BC) is a microbial polysaccharide biopolymer created by nanofibrils made mainly by Gluconacetobacter xylinus, Acetobacter hansenii. It has a similar chemical construction presented by plants [76]. BC has individual characteristics broadly used in medical, cosmetics, industrial, and electronic purposes, especially in biomedical science and food packaging, because its three-dimensional nanofibrillar networks have excellent mechanical and barrier performance, water-holding ability, and structure-forming potential [77,78]. Bacterial cellulose has a unique construction of nanofibrils, making a nanostructured network characterized by high pureness (compared to plant cellulose, BC is free of elements such as hemicellulose and lignin) [79, 80]. The biocompatibility, excellent crystalline features, high mechanical stability, and high-water absorption capacity in the wet state of BC can increase the strength of the biocomposite films. BC could also absorb moisture, increase O_2_ permeability and water vapor permeability (WVP), and the thermal stability of films. Furthermore, the United States Food and Drug Administration has classified BC as safe (GRAS) since 1992 [80–82]. In Asian nations, using “traditional” fermentation techniques, BC is constructed and sold as a high-fiber food and low-calorie dessert [83]. Overall, BC can be quickly prepared into nanofibrils, microfibrils, and nanocrystals and can be utilized to fabricate nanocomposite films [76]. Because bacterial cellulose nanofibers (BCNFs) have limited antibacterial and antioxidant activity, they are rarely used as a food packaging film or wound treatment [78].

(2) Carboxymethylcellulose (CMC), an anionic cellulose derivative, which because of its availability, cheap cost, biodegradability, hydrophilic, nontoxic, and renewable properties, has gained special attention for enhancing bio-based films. CMC can be synthesized with the catalyzed cellulose reaction with monochloroacetic acid [84]. As an edible film and water-soluble polymer, it is extensively utilized in food processing as stabilizers and thickeners in different foods. It can create highly original and flexible biodegradable films with acceptable mechanical characteristics. It has also been given the label of “generally recognized as safe” (GRAS) [66]. CMC is a water-soluble cellulose derivative used as an additive in pharmaceutical and food applications. It has an essential role in fabricating many support materials for many enzymes such as isoamyl lactase, *a*-amylase, lipase, and polyphenol oxidase. Furthermore, CMC has been described as a valuable adsorbent to remove dyes, owing to the comparatively abundant carboxyl groups [85, 86].

(3) Cellulose nanowhiskers (CNWs) are usually obtained by hydrolysis techniques from natural cellulose-based materials such as bacteria, plants, and sea creatures. Its size varies from a few nanometers to several tens of nanometers in diameter and 100 nm [87]. CNW has been employed in the creation of advanced valuable materials for purposes such as biomedical, optical, structural, tissue engineering [88], drug delivery [89], energy storage [90], electronic devices [91], wastewater treatment [92,93], coating additives, molecular biology, paper production, cosmetics, composite materials [94,95], and food packaging [96,97]. CNWs have attracted attention because, as a nanostructure, they present low density, extensive surface area, high mechanical stability, high modulus of elasticity, low thermal extension coefficient, large aspect ratios, optical transparency, unique morphology, smooth of production, and chemical reactivity [87, 88, 98–102]. Several desired qualities of the CNW include abundance, repeatability, low density, low cost, considerable and varied surface area, biocompatibility, and biodegradability, which have seen much application in the polymer composite reinforcement [103]. When combined with polymeric film materials, cellulose nanowhiskers (CNWs) with high aspect ratios improve the film features [104]. These characteristics make it an excellent biopolymer to employ in the food packaging [96,97].

#### 3.1.4. Chitosan

Chitosan is a chitin-based polymer with a linear structure ordered by deacetylated and acetylated units [70]. After cellulose, chitosan is the second most abundant polysaccharide on the planet, and it may also be found in some fungal cell walls [105]. Chitosan gives an extensive range of purposes, including the purification of water and beverages, cosmetics and pharmaceuticals, biotechnological and agricultural applications, and food packaging [106]. Chitosan is nontoxic and biodegradable, and possesses several natural properties, including enhanced strength and elongation. On the one hand, chitosan films exhibit outstanding mechanical properties and selective permeability to gases (CO_2_ and O_2_).

On the other hand, its hydrophilic nature has a poor moisture barrier, limiting its applications [107, 108]. The degree of deacetylation, the molecular weight, the number of acetyl groups on the main chain, and the kind of acid used for protonation all affected chitosan solubility due to the amino groups being soluble in weak acid solutions lower than pH 6.0, unlike chitin. Aside from solubility, chitosan's molecular weight might affect the film's quality, including optical properties, brittleness, and elasticity [109, 110]. The USFDA has authorized chitosan as a food supplement [111]. Food safety against microbial deterioration, the creation of edible biodegradable films, condensation of proteins and lipids from wastewater, enhancing gelation in fisheries goods, and deacidification of fruit juice are only a few items for chitosan in the food applications [112]. Chitosan is an antibacterial biopolymer that may be used as an antimicrobial agent and a biopolymer substrate. Because of their superior antibacterial properties, chitosan films are acknowledged as eco-friendly food packaging. They have a long shelf life and keep food fresh [113]. Moreover, in contrast to water, in acidic solutions, it dissolves easily [70].

#### 3.1.5. Polylactic Acid (PLA)

Polylactic acid (PLA) is one of the most promising and environmentally friendly polymers due to its exceptional physical and chemical features: renewability, biodegradability, and biocompatibility. PLA is made from renewable sources such as maize starch (in the United States and Canada), cassava roots or starch (in Asia), or sugar cane (in the rest of the globe) [114]. Corn-derived PLA is biodegradable, making it ideal for nutritional uses. According to the USFDA (United States Food and Drug Administration), the PLA has considered an entire safe status (GRAS) [115]. In 2010, PLA accounted for the enormous consumption volume globally compared to other bioplastics [116]. On the other hand, PLA has a significant permeability to gas and vapor, limiting its use in food packaging for short-term storage [117]. Given that PLA is nontoxic, noncarcinogenic, biocompatible, hydrophilicity, water-soluble, and chemically steady, it is typically combined with other polymers.

#### 3.1.6. Xanthan Gum

Xanthan gum is a pentasaccharide. It is an extracellular heteropolysaccharide with high molecular weight created by Xanthomonas campestris [118, 119]. Xanthan gum consists of D‐glucose, D‐mannose, and D‐glucuronic acid units and in the proportion of 2 : 2 : 1 [120, 121]. Approximately half of the terminal D-mannose is connected to pyruvyl residues. It has converted to one of the most effective hydrocolloids due to its high ability, especially in high salt, acid, and shear stress. It is steady over a broad pH range [122, 123]. Xanthan is accepted as a food component by the USFDA [124]. Xanthomonas campestris is a Gram-negative aerobic rod bacterium that is one of the most lucrative industrial microbial hydrocolloids used as a thickening factor and stabilizer in the food preparation [125, 126]. The mechanical characteristics and moisture adsorption of cassava starch films have been affected by the xanthan [127]. Xanthan gum has a pseudoplastic rheological performance in an aqueous environment employed in film production. It disperses well in water at any temperature, and pH or temperature does not affect its viscosity [123, 127]. The xanthan gum boosted film traction stability but made the matrix less flexible. Xanthan is proper for food packaging because of its ability to adjust viscosity. However, more research is needed to see whether it can fully stand alone as a food packaging source [71]. Gelatin films combined with xanthan gum present a transparent film with extreme UV light resistance, low solubility, moisture content, low water vapor permeability (WVP), increased mechanical characteristics, and thermal stability [127]. Presently, mixing with different biopolymers is recommended to produce an excellent food package. The expensive production is another critical problem facing its production [71].

#### 3.1.7. Gellan Gum (GG)

Gellan gum (GG) is an anionic, water-soluble, high-molecular-weight, and deacylated exocellular polysaccharide that is secreted from bacteria belonging to the Sphingomonas genus and Sphingomonas paucimobilis (earlier *Pseudomonas* elodea) [128–131]. First isolated in 1979, these bacteria have a helix-forming and gelling ability produced as a fermentation product. GG is currently manufactured in vitro by a simple fermentation method, bypassing batch-to-batch availability associated with the biopolymers [132]. GG is made up of a repeated linear chain of tetrasaccharide units and consists of a linear chain of tetrasaccharide parts (L-rhamnose, D-glucose, and D-glucuronic acid) in a 3 : 1 : 1 molecular ratio, containing one carboxyl side group [120], commercially available under many trade names (e.g., Phytagel^TM^, Gelrite^®^, and Kelcogel^®^) [128, 132, 133]. After FDA approval as a food additive in 1992, this polysaccharide is extensively exploited in the food industry as a thickening factor or emulsion stabilizer to improve food quality [129, 132, 134]. Gellan gum hydrogel films, because of the biocompatibility and low cytotoxicity, has widespread use in pharmacology (implant for insulin delivery, and nasal and ophthalmic drug delivery agent), cell carrier, an anti-adhesion barrier, a guided bone-regeneration material, tissue engineering, and wound dressing materials [129, 135, 136]. Polymer negative groups interact with divalent or multivalent counter ions to form a more substantial, thick hydrogel network. GG shows unique film-forming, biodegradable, biocompatible features, as well as an excellent drug release kinetics [128]. The gelation conditions bring about a wide diversity of textures and mechanical properties [135]. Furthermore, gellan gum is in the coil form at high temperatures or in acid circumstances, presenting higher heat and acid stability for active compounds and probiotics [128].

### 3.2. Carbohydrate Polymers Based on CuONPs in Food Packaging

Many researchers have employed silver, zinc oxide, and titanium dioxide nanoparticles to increase the water vapor barrier, mechanical properties, and antibacterial properties of films [137–140]. In addition to these nanoparticles, copper oxide nanoparticles (CuONPs) have attracted researchers due to their novel features such as a high surface-to-volume ratio, barrier to visible and UV light, low water vapor preliminary, cheaper element compared to other nanoparticles, increased mechanical characteristics in synthetic polymers, and nontoxicity [141, 142].

With the decrease in size, the CuONP properties increase, and finely dispersed bioactive CuONPs are predicted to have a significant disinfectant action [143]. Because of the tiny size, unbound Cu ions can interact with bacterial membranes more effectively.

Copper oxide nanoparticles (CuONPs) are generated by combining copper salts (copper sulfate, copper acetate, and copper chloride) with reductants (NaOH and ascorbic acid) to form CuONPs [143]. With the decrease in size, the CuONP properties increase, and finely dispersed bioactive CuONPs are predicted to have a significant disinfectant action. Because of the tiny size, unbound Cu ions can interact with bacterial membranes more effectively [144]. Numerous techniques have been explained by the association of CuO nanoparticles, antibiotics, and natural active compounds into BCNF-based films to improve their antibacterial and antioxidant activities [145, 146]. Copper oxide nanocomposite films are interesting for active packaging and reveal a higher antimicrobial effect against Gram-negative (*E.coli*) and Gram-positive (*S. aureus*) food spoiling microorganisms [145, 147, 148] and also antioxidant activities such as DPPH and ABTS in AFP [149]. As a result, various studies on the effects of CuONPs on biopolymer films have been carried out.

### 3.3. Physical and Mechanical Characteristics of Carbohydrate Polymers Based on CuONPs

The mechanical features of materials principally depend on their performance in critical situations such as temperature, cooling rate, heating, deformation, applied force, and deformation rate [150]. Biopolymer composites' mechanical properties depend entirely on the biopolymer and its chemical construction, morphology, molecular weight, molecular orientation, crystallinity, copolymerization, plasticization, crosslinking filler type, and concentration, which determine expected functionalities as well as the importance of the material [150]. In general, the mechanical properties of composite films created by the blending process are determined by the interactions between the compounds and their miscibility and intermolecular cooperation between polymer chains [151]. There are a different variety of mechanical properties that improve in understanding the material and its characteristics, including tensile strength (TS), elongation at break (EB), elastic modulus (EM), Young's modulus (YM), thickness, yield stress, Poisson's ratio, storage, creep, and recoverable compliance [152].

Mechanical properties such as knock and tensile strengths considerably improve composites when bio-derived reinforcement materials are acclimatized into the biopolymers [153, 154]. The polymer part in biocomposites helps the composite exhibit better mechanical properties in molecular weight, chemical components, morphology, and processing method, which helps impart desired functionalities to the material [155]. Meanwhile, imbued with bio-derived nanoparticles and nanofibers, the ensuing biopolymers show development in moduli features, gas permeability, heat deformation temperature, decomposition, and, in individual, the tensile performance of the resulting component [156, 157]. For improved eco-friendly and economical, biopolymer nanocomposites encourage researchers to produce novel products compatible with various food packaging applications [158]. A particular portion of research devotion has been set on combining biopolymers with CuO nanoparticles that contain compounds such as cellulose, carboxymethylcellulose (CMC), and bacterial cellulose (BC), starch, agar, xanthan gum, gellan gum, and chitosan[158].

Shankar and his colleagues [142], combined copper nanoparticles with five types of biopolymers (agar, alginate, carrageenan, chitosan, and CMC). They investigated its mechanical properties, including thickness, TS, EB, EA, and TGA thermal stability (Table 2).

The alginate-CuONPs and CMC-CuONPs have the lowest and highest thickness compared to other films. The thickness of the biopolymer films was raised after the addition of CuONPs. The large number of CuONPs in the composite films caused the thickness of the films to expand. Biopolymer films” tensile strength (TS) varied based on the polymer type. Compared to other films, the lowest and maximum TS owing to agar-CuONPs and carrageenan-CuONPs were 38.6 MPa and 55.5 MPa, respectively. The TS and EM significantly improved in the chitosan, alginate, and CMC-based films. However, in the case of agar and carrageenan-based films, the TS rose little. The elastic modulus (EM) of CMC-CuONPs was the lowest with 1.08 GPa, and chitosan, carrageenan, alginate, and agar nanocomposite films were the highest at 1.77, 1.62, 1.34, and 1.21 GPa, respectively. The nanocomposite films presented a more favorable TS and EM value than neat polymer films. On the opposite, the flexibility (EB) of the biopolymer films declined after incorporating CuONPs. The highest percentage of elongation at break (EB) refers to CMC, chitosan, agar, alginate, and carrageenan-CuONPs, with 54.2%, 21.8%, 20.3%, 18.4%, and 11%, respectively (Table 2).

Thermal stability was measured using the thermogravimetric analysis (TGA) method. For all of the films, the first thermal degradation started at 80°C. The evaporation of moisture caused this phase of thermal deterioration. The second stage differed depending on the kind of biopolymer film; this process is linked to the degradation of glycerol and biopolymers. For the CMC films, chitosan, agar, carrageenan, and alginate films, the highest temperatures for the second stage degradation were 277, 270, 248, 229, and 218°C, respectively. When CuO nanoparticles were mixed, the highest degradation temperatures were altered to 277°C, 264°C, 259°C, 253°C, and 219°C, respectively. After adding CuONPs, the start temperatures for thermal disruption of carrageenan and agar films increased, decreasing in chitosan-based nanocomposite films. This might be owing to the copper ionic forms' configuration. However, there was no difference in the case of alginate- and CMC-based films. The film's mechanical properties are primarily determined by the polymer chains' thickness, density, concentration, and intermolecular and intramolecular interactions [164].

In a different study, Peighambardoust and his colleagues [159] investigated the role of starch biopolymer with silver, zinc, copper nanoparticles, and a combination.

This study assessed the physical and mechanical properties of starch-based nanocomposite films combining Ag, CuO, and ZnO as single NPs or a mixture of NPs (Table 2).

The mechanical properties of nanocomposite films are influenced by two significant factors: to acquire the nanocomposite films' excellent mechanical properties, sufficient contact between the surface of nanoparticles and the biopolymer matrix is necessary. Moreover, the biopolymer matrix must have a homogenous dispersion of all nanoparticles [165]. Film samples such as starch-Ag, starch-ZnO, starch-CuO, and starch-Ag-ZnO-CuO of NPs were chosen for mechanical analysis, that is, thickness, TS, EB, and YM. A micrometer was used to measure the thickness of each film, which was 120 ± 5 *μ*m. ZnO, CuO, and Ag starch films were found to have tensile strengths of 7.50, 6.03, and 4.17 MPa, respectively. The tensile strength of the biopolymer containing starch, Ag, CuO, and ZnO film, on the other hand, was lower than CuONPs and higher than Ag NPs. At 2wt.% of each nanoparticle, the TS of starch-ZnO film outperformed starch-Ag and starch-CuO films. The strong molecular interaction between starch chains and ZnO-NPs appears to be the source of this issue [122] (Table 2).

They also confirmed that the EB status of the starch-Ag-ZnO-CuO nanocomposite film was higher than other film samples. The starch film had the highest EB, with 98%, followed by starch-Ag-ZnO-CuO, starch-CuO, starch-ZnO, and starch-Ag nanocomposite films with 76.67%, 65%, 55.67%, and 42.67%, respectively (Table 2). The force required to extend the film was increased by biopolymer chains, resulting in a lower EB value. The mechanical strength of the resultant films is influenced by the interfacial interactions between nanofillers and their dispersion in the biopolymer matrix.

Young's modulus (YM) is another factor for evaluating mechanical strength. Peighambardoust et al. showed that starch-ZnO has the highest YM with 25.44 MPa, starch-CuO, starch-Ag-ZnO-CuO, starch-Ag with 22.82, 19.87, and 14.05 MPa, respectively. These results showed that the starch-CuO film has a more potent ability, great flexibility, high YM, and TS than starch-ZnO and starch-Ag and can be a good film for AFP.

In another study, Vasile and his colleagues [160] demonstrated ZnO's effect: Cu/Ag nanoparticles on the properties of plasticized PLA were examined in terms of architecture. Melt blending processing techniques provided polylactic acid (PLA) samples with embedded Cu-doped ZnO powder functionalized with Ag nanoparticle composites (PLA/ZnO: Cu/Ag). They examined thermal stability and mechanical properties such as thickness, TS, EB, and YM with neat PLA and four different PLA nanocomposite films (0%–1.5 wt.%).

The thickness of neat PLA and each biopolymer nanocomposite film PLA/ZnO : Cu/Ag (0%–0.5%–1% and 1.5%) reported lower than 200 micrometers (Table 2). Compared to other nanocomposite films, the neat PLA had the highest tensile strength, with 69.28 MPa. Among the PLA/ZnO: Cu/Ag films, the 1wt.% had the maximum TS with 48.39 MPa, 1.5, 0.5, and 0 wt.% with 47.28, 45.32, and 44.81 MPa, respectively. The addition of ZnO: Cu/Ag nanoparticles into plasticized PLA has slowly increased the effect of the TS at the break with the highest value of 48–47 MPa for ZnO: Cu/Ag 1 or 1.5% (Table 2). This is likely due to a homogenous dispersion of ZnO: Cu/Ag NPs into the PLA matrix and nanoparticles' high aspect ratio that reduces the chain movements. A similar advancement in PLA film mechanical properties of the nanocomposites strengthened with Ag-Cu composite or ZnO nanoparticles [166, 167].

The elongation at break (EB) is another essential mechanical characteristic in food packaging. The lowest flexibility refers to a neat PLA with 2.14%. On the other hand, PLA nanocomposite films had a higher EB than neat PLA. The PLA/ZnO : Cu/Ag 0 wt.% has the highest EB with 3.30%, followed by 0.5, 1, and 1.5 wt.% with 2.78%, 2.67%, and 2.61%, respectively (Table 2). These results presented that the EB decreased with the increase in the NP content. However, compared to the neat PLA, nanoparticles help in increasing the flexibility of films.

On the opposite of EB, neat PLA has the maximum YM value compared to PLA/ZnO: Cu/Ag films, and the YM increased with the increase in the nanoparticle content. Vasile et al. explained that neat PLA has the highest YM with 4048 MPa. Among the PLA/ZnO: Cu/Ag films, PLA/ZnO: Cu/Ag 1% films have the highest YM with 3058 MPa, followed by 1.5, 0.5, and 0 wt.% with 3010, 2934, and 2898 MPa, respectively.

It is recognized that PLA below the functional processing conditions displays a low crystallinity. Due to the intrinsic slow crystallization rate, it is an amorphous shape, restricting its applications in the automotive and packaging fields. The cooperative influence of plasticizers and nucleating factors in the crystallinity enhancement was determined by investigating the thermal and mechanical characteristics of the PLA nanocomposites [168].

This study measured thermal stability by the differential scanning calorimetry (DSC) technique. The DSC curves of PLA nanocomposite films displayed a glass transition temperature (is described as the temperature at which 30–50 carbon chains began to move) [169], an excellent visible exothermal cold crystallization process, and the endothermal melting process as a double peak, apparently due to diverse sorts of crystallites with varying lamellar thickness or reorganization during melting. The glass-liquid transition, also known as the glass transition, is the gradual and reversible transition in amorphous materials from a hard and moderately brittle “glassy” state to a viscous or rubbery state when the temperature is increased [170]. The DSC curve of neat PLA is recognized as a transition at 57.5°C associated with the glass transition (Tg) relaxational process. Among the PLA nanocomposite films, PLA/ZnO: Cu/Ag 1% has the highest Tg with 48.5°C compared to other films, which have 47.5, 46.4, and 44°C related to 1.5, 0.5, and 0 wt.%, respectively (Table 2).

Mary et al. [161] investigated the tensile strength of chitosan-attached cellulose (CAC), copper-bound chitosan-attached cellulose (CBCAC), and CuONP chitosan cellulose (NCLCAC) biopolymers. The results of TS analysis have been well described. The TS of CAC, CBCAC, and NCLCAC biopolymers was determined to be around 30.58, 59.77, and 38.88 MPa, respectively (Table 2).

The presence of Cu (II) ions that bond firmly with the nitrogen atoms of the amino group of chitosan chains in the CBCAC biopolymer explains why the CBCAC biopolymer has more TS than the conventional CAC biopolymer. Plain and CuONPs-chitosan-cellulose (NCLCAC) biopolymers were shown in DSC thermograms. The glass transition temperature (Tg) for these samples is almost 70°C and 150°C, respectively, indicating the thermal stability of biopolymers verified thanks to the CuONP coating (Table 2).

In one study, [162], the films' thickness and DSC were measured. The thickness of the chitosan and CuNP-chitosan films was 46 *μ*m and 53 *μ*m, respectively. The nanocomposite film, which contained 0.17% colloidal CuNPs, increased the thickness of the chitosan film by 15%. The DSC curves of films showed a primary endotherm change to lower temperature regarding CS powder for chitosan and CuNPs-chitosan films that happened above a temperature range (70–140°C). This endotherm refers to water loss and describes the energy required to evaporate the water in the films. The moisture retentivity of chitosan film was affected when colloidal CuNPs were added. The composite film's expected energy is much lower, maybe, because of the loss of the hydrogen bonding caused by the sterical barrier of colloidal CuNPs. Compared to the chitosan film, the composite film had a weaker exotherm at 228°C.

The hopeful mechanical characteristics of polymer composite films make it possible to use them in food packaging and technology. Saravanakumar et al. [149] produced four combination films with cellulose, sodium alginate (SA), and CuO nanoparticles with various food packaging concentrations for food packaging, and showed the thickness as mechanical characteristics but not reported the TS, EB, EM, YM, and thermal stability. In this study, the thickness of sodium alginate (1%)-CuONPs (1 mM), sodium alginate (3%)-CuONPs (1 mM), sodium alginate (3%)-CuONPs (5 mM), and cellulose nanowhisker (CNW) (0.5%)-SA (3%)-CuoNPS (5 mM) were determined to be around ∼58, ∼62, ∼67, and ∼71 *μ*m, respectively. This issue explained that the thickness of the polymeric films rose with the CuONPs, and the SA concentration increased. Similar effects were recognized with cellulose nanocomposite films embedded with ZnO-NPs [138, 171] (Table 2).

In a study, Oun et al. [172] investigated carrageenan films prepared by combining with ZnO, CuONPs, and their combination. The mechanical characterization was evaluated based on thickness, TS, EB, and EM for carrageenan-KCl, carrageenan-KCl-ZnO (1%), carrageenan-KCl-ZnO (0.5%)-CuO (0.5%), and carrageenan-KCl-CuO (1%) (Table 2). According to the thickness, it was concluded that the nanocomposite film containing zinc oxide was less thick than copper oxide nanoparticles. However, the carrageenan-KCl-ZnO (0.5%)-CuO (0.5%) composite had a thickness between these two nanocomposite films.

The neat carrageenan had the highest TS and EM, with 55.2 MPa and 3.37 GPa compared to other films. The TS for carrageenan-KCl-ZnO (0.5%), carrageenan-KCl, carrageenan-KCl-ZnO (0.5%)-CuO (0.5%), and carrageenan/KCl-CuO (1%) was 48.2, 44.9, 32.9, and 30.4 MPa, respectively. The EM was measured for carrageenan-KCl-ZnO (0.5%), carrageenan-KCl, carrageenan-KCl-ZnO (0.5%)-CuO (0.5%), and carrageenan-KCl-CuO (1%) with 2.37, 2.24, 0.89, and 0.88 GPa, respectively.

After the fillers were added, the TS and EM of the neat carrageenan film decreased dramatically. The recrystallized KCl in the polymer matrix caused fissures in the polymer matrix, resulting in a decrease in the film's TS and EM. The EB films altered depending on nanofiller types, although their performance was contrary to the TS and EM. The carrageenan-KCl-ZnO (0.5%)-CuO (0.5%) and neat carrageenan had the highest and lowest EB compared to other films with 18.2% and 6.6%, respectively. After that, carrageenan-KCl-CuO (1%), carrageenan-KCl-ZnO (1%), and carrageenan-KCl with 15.9%, 10.3%, and 6.7% respectively (Table 2).

Using thermogravimetric analysis, the thermal stability of carrageenan-based films was investigated (TGA). Carrageenan-KCl, carrageenan-KCl-ZnO (0.5%)-CuO, and carrageenan-KCl-ZnO (0.5%)-CuO were all decomposed at 210°C. However, the carrageenan-KCl-CuO (1%) film lowered to 200°C. In contrast, it expanded to 228°C in the carrageenan-KCl-ZnO (1%) film. The maximum breakdown temperatures (*T*_max_) of the films were 228°C, 222.5°C, 222.5°C, 252°C, and 228°C for carrageenan, carrageenan-KCl, carrageenan-KCl-CuO (1%), carrageenan-KCl-ZnO (1%), and carrageenan-KCl-ZnO(0.5%)-CuO (0.5%), respectively, as determined by the peak temperature in the DTG curve (Table 2).

In another study conducted by Hasheminya and his colleagues [163], they investigated the kefiran-CMC biopolymer's mechanical properties by increasing copper oxide nanoparticle concentration. This study combined three concentrations of 1%, 1.5%, and 2% copper oxide nanoparticles with the kefiran-CMC biopolymer. According to the findings, the thickness of the biopolymers rose as the concentration of copper oxide nanoparticles increased. The highest thickness was due to kefiran-CMC-CuONPs (2%) with 117 *μ*m followed by CuONPs (1.5%) and CuONPs (0.5%) with 110 and 107 *μ*m, respectively (Table 2). With increasing copper oxide nanoparticle concentration, the contact angle of the films rose as well. From maximum to minimum, the contact angle was due to kefiran-CMC-CuONPs (2%), kefiran-CMC-CuONPs (1.5%), and kefiran-CMC-CuONPs (0.5%) with 43.79°, 40.12°, and 37.38°, respectively.

Increasing the angle of films in hydrophobic environments is associated with decreased hydrophobic compounds and decreases in hydroxyl groups in the biopolymers [173]. One of the reasons could be the hydrophobicity of copper oxide nanoparticles [174].

Like the contact angle and thickness, the tensile strength of the copper oxide nanoparticles increased. The highest TS was due to kefiran-CMC-CuONPs (2%) with 4.48 MPa, followed by kefiran-CMC-CuONPs (1.5%) and kefiran-CMC-CuONPs (0.5%) with 4.12 and 3.62 MPa, respectively (Table 2). This is due to hydrogen bonding between the hydroxyl groups of the copper oxide nanoparticles and the kefiran-CMC biopolymer. It is in line with the study by Rao et al. [175], which investigated the role of copper oxide nanoparticles in polyvinyl alcohol composite films. The composite film's increased tensile strength was credited with bonding in the hydroxyl groups of polyvinyl alcohol and copper oxide nanoparticles. Contrary to the tensile strength, contact angle, and thickness, the elongation at break decreased with the increasing concentration of nanoparticles. The lowest EB was due to kefiran-CMC-CuONPs (2%) with 60.74% followed by kefiran-CMC-CuONPs (1%) and kefiran-CMC-CuONPs (0.5%) with 64.41% and 71.78%, respectively. Shankar et al. [142] reported the same result investigated on alginate, chitosan, and carrageenan, which we described earlier. Thermal analysis in this study showed that both heat transfer steps increased with the increasing concentration of copper oxide nanoparticles in the biopolymer. The film's glass temperature (Tg) reached 25.43, 44.81, and 83.13°C for 0.5%, 1%, and 2% concentrations, respectively, in the upper heat transfer phase. The lower thermal transition stage of the control film was −44.55°C and was reported in films with 0.5%, 1%, and 2% CuONP concentrations at −6.16, 10.72, and 37.8°C, respectively (Table 2).

The above studies show that copper nanoparticles can combine with various biopolymers, including starch, chitosan, agar, carrageenan, alginate, polylactic acid, cellulose (CMC, CNW, BC), and other polymers. With that, thickness, tensile strength (TS), and film flexibility are increased.

### 3.4. Food Barriers of CuO-Carbohydrate Films

Managing water vapor and gas emission rates is essential to reach adequate quality, safety, and shelf life for moisture-sensitive foods. Beyond packaging, high-performance films with high elasticity, optical clarity, mechanical strength, thermal stability, biodegradability, and gas barrier properties are needed for various applications [176]. Carbohydrates, especially polysaccharides, have high-quality material for excellent mechanical features, but it has a weak water vapor transfer barrier. On the other hand, combining lipids provides acceptable water vapor barrier qualities; however, the resulting films are frequently opaque, brittle, and unsteady and have a waxy taste [177]. Nanocomposites are combined in the materials' biopolymer matrix due to their large surface area, favoring the filler-matrix cooperation and activity.

Furthermore, the nanocomposites reinforce the act as little barriers for gases by complicating the material [14]. Plastics are approximately penetrable to small molecules such as vapors (gases such as O_2_, CO_2_, N_2_, and H, water, and organic vapors) or liquids. Water vapors and gases are two principal penetrable considered in packaging purposes. These composites may transfer from the inner or outer environment through the polymer package wall, following a constant change in product property, reducing the shelf life [178,179]. Most fresh foods are spoiled due to high humidity [180].

#### 3.4.1. Water Vapor Permeability (WVP)

Water vapor permeability (WVP) can estimate water vapor barrier features, which uses the differential pressure and thickness of the packaging material and shows the quantity of water infiltrated per unit area and time (kg/m *s* Pa). Fresh food items, such as vegetables, require water vapor barrier properties to avoid dehydration. In contrast, other foods, such as bread or dry foods, require water vapor barrier features to prevent moisture absorption from the environment, which is critical [179]. Another water vapor permeability factor is the water vapor transmission rate (WVTR) used in bread packaging, as expressed in cc.m^−2^. s^−1^ (or gm^−2^.day^−1^) [55].

#### 3.4.2. Oxygen and Carbon Dioxide Permeability

Limiting gas transfer into food packaging for longer shelf life is a significant challenge for packagers. The transfer of oxygen into a beer bottle can cause it to be stale; similarly, its presence in the soda bottle can reduce its shelf life [181]. The effect of oxygen on food and container shelf life is complex. Oxygen is essential for certain foods to prevent the matrix from rapidly deteriorating. For instance, there is respiratory metabolism in fresh fruits and vegetables. However, it is not difficult to say that oxygen is considered the main enemy of many food products [49,182]. This is a significant cause of chemical degradation for several matrices because it is associated with harmful reactions.

Due to the quick oxidation of lipids and vitamins in the food or by improving microorganisms such as aerobic bacteria, yeasts, and molds, the presence of oxygen in food packaging causes rapid food loss [49,182]. The presence of oxygen in packaging causes additional adverse effects on enzymatic systems. Using oxygen as a second substrate for enzymatic function may cause water and beverage discoloration. Lipases are lipolytic enzymes that hydrolytically stimulate the release of free fatty acids from triglyceride molecules. The presence of oxygen may catalyze the plant's browning tissues by polyphenol oxidase, [183]. Due to oxygen, all of the above destructive reactions can lead to discoloration or taste of the food, leading to food poisoning, slowing food life, and endangering consumers' health and safety.

As a result, one of the essential aims in the food packaging sector is to manage the oxygen inside the package and its exposure to the food [182]. The presence of carbon dioxide gas at the right concentration can inhibit or slow down bacteria's growth, thereby helping the freshness in the food packaging [184]. The antimicrobial role can also act as an antioxidant and prevent food from maintaining oxidation [185]. On the other hand, depending on the type of food (such as yoghurt) and its storage temperature, it can accumulate a lot of carbon dioxide, which results in lost quality of the food product and its packaging [55]. To this end, making a biopolymer nanocomposite with suitable barrier properties for packaging, preserving food quality, and reducing plastic consumption is one of the urgent needs today. In recent years, research on the joint role of copper oxide nanoparticles and biopolymers has been undertaken, which we shall examine in this section.

Shankar and his colleagues [142] combined copper nanoparticles with five different biopolymers (agar, alginate, carrageenan, chitosan, and CMC). They investigated its barriers to properties, such as WVP, and used UV light for optical transparency. The films were exposed for 48h at 25°C and 50% RH before determination. Among the films, the carrageenan film showed the highest WVP value (1.84 ± 0.26 × 10^−9^ g m/m^2^.Pa.s) followed by alginate, CMC, agar, and chitosan films with 1.74 ± 0.08, 1.72 ± 0.34, 1.54 ± 0.18, and 1.09 ± 0.23 × 10^−9^ g m/m^2^.Pa.s, respectively (Table 3). The WVP of the biopolymer-based films reduced significantly following the incorporation of CuO nanoparticles. In this regard, the highest WVP value for nanocomposite films refers to CMC-CuONPs with 1.40 ± 0.08 × 10^−9^ g m/m^2^.Pa.s. followed by alginate-CuONPs, carrageenan-CuONPs, agar-CuONPs, and chitosan-CuONPs nanocomposite films with 1.35 ± 0.17, 1.19 ± 0.08, 1.18 ± 0.06, and 1.03 ± 0.17 × 10^−9^ g m/m^2^.Pa.s, respectively. Water vapor impermeable metallic nanoparticles (MNPs) formed an indirect conduit for water vapor diffusion through the polymer matrix, resulting in a decrease in WVP [186] (Table 3).

The percentage of light transmission in UV and visible areas was calculated by applying a spectrophotometer, and the results were presented. Compared to nanocomposite films, the neat carbohydrate-based polymer films were extremely transparent. In this regard, at visible light (*T*_660_), the carrageenan film showed the highest light transmission with 89.6 ± 0.6%. After that, agar and chitosan had the same light transmission with 89.1 ± 0.5 and 89.1 ± 0.3%, respectively, followed by alginate with 88.7 ± 0.3 and 87.7 ± 0.9%, respectively. At the same visible light (T_660_), the percentage of light transmission in the nanocomposite films decreased. The chitosan-CuONPs was the highest transmittance light with 75.4 ± 0.9% followed by carrageenan-CuONPs, alginate-CuONPs, agar-CuONPs, and CMC-CuONPs with 15.8 ± 0.9%, 14.3 ± 0.4%, 13.9 ± 0.7%, and 9.63 ± 0.5%, respectively (Table 3).

At the wavelength of UV light (T_280_), similar results were obtained with visible light results, meaning that at ultraviolet wavelengths, the transmission of light in nanoparticle-free biopolymers was higher than that of those with copper nanoparticles. The carrageenan film was the highest light transmission at wavelengths of UV light, with 73.9 ± 1.1%. followed by alginate, CMC, agar, and chitosan with 67.0 ± 1.0%, 58.3 ± 2.5%, 49.1 ± 1.2%, and 8.8 ± 6.0%, respectively (Table 3). At the same wavelength, the light transmission in nanocomposite films is reduced. In this term, carrageenan-CuONPs with 9.3 ± 1.1 had the maximum transmission, followed by alginate, agar, CMC, and chitosan nanocomposites with 6.2 ± 0.4, 3.75 ± 0.3, 1.4 ± 0.1, and 0.1 ± 0.0, respectively. Compared to other biopolymer films, the transparency of chitosan-based films decreased only a little. This could be due to the acetic acid in the chitosan-based composite film converting CuONPs to copper ions. Their result indicated that the MNP performed an essential role against both UV and visible lights of all the films and decreased significantly after incorporating CuO nanoparticles [142]. This chiefly depended on UV light absorption by nanoparticles dispersed in the film matrix [187]. Like this report, Shankar et al. also observed the lower transmission of UV light in the Agar-CuO nanoparticles [143]. The combination of copper nanoparticles with various biopolymers reduces the transparency of films due to the surface plasmon resonance (SPR) of CuO nanoparticles [188].

Therefore, WVP should be as low as possible to determine the lowest moisture content in the food packaging and the surrounding atmosphere to keep the quality [189, 190]. In this regard, a study recognized the role of starch biopolymer with silver, zinc, copper nanoparticles, and a combination to assess the WVP and water solubility films. Peighambardoust et al. [159] showed WVP of the neat starch film and nanocomposite films combining single and a combination of CuO, ZnO, and Ag nanoparticles at varied concentrations. Before calculation, the films were exposed for 48h at 25°C and 50 ± 5% RH. Regardless of the nanoparticles used in starch, this study clearly showed that by adding nanoparticles, the starch film nanocomposites' water vapor content decreased compared to that of a pure starch film. This property increased with increasing concentrations of copper oxide, zinc oxide, and silver nanoparticles. By increasing the %weight of copper oxide nanoparticles in the starch matrix, water vapor permeation becomes more severe, reducing WVP. The same is valid for the zinc and silver nanoparticles [172].

On the other hand, filling the spaces in the starch biopolymer matrix and forming hydrogen bonds in the starch macromolecular chains reduce water penetration and, ultimately, water vapor permeability in the film [190]. However, WVP was lower in the St-Ag-ZnO-CuO film at equal concentrations than all other films, reportedly due to the uniformity and dispersion of copper oxide, zinc oxide, and silver nanoparticles.

Water solubility (WS) is another crucial barrier feature in biopolymers. In this study, nanocomposites containing a single or a combination of zinc oxide, silver, and copper oxide nanoparticles were investigated. Regardless of the nanoparticles used in the starch matrix, the metal nanoparticles' composition in the starch matrix significantly reduced its solubility in water, increasing nanoparticle concentration. Compared to zinc and silver nanoparticles, copper oxide nanoparticles did not perform well in this respect. This might be for water solubility, as compounds containing copper oxide nanoparticles increase; on the other hand, they reduce hydrogen bonds [191].

Vasile et al. [160] demonstrated ZnO's effect: Cu/Ag nanoparticles on plasticized PLA properties. The water vapor and gas permeability transmittance rate of the developed bionanocomposites is utterly described. The water vapor transmittance rate of neat PLA was 15.94 g·m^−2^ day^−1^. Among the PLA/ZnO: Cu/Ag films, the 1wt.% had the highest water vapor transmittance rate, with 18.72 g·m^−2^ day^−1^ compared to other nanocomposite films followed by 1.5, 0, and 0.5 wt.% with 15.6, 13.70, and 11.35 g·m^−2^ day^−1^, respectively (Table 3).

The gas permeability transmittance rate was reported for neat PLA, 0, and 0.5 wt.% of PLA/ZnO: Cu/Ag films. The transmittance rate of carbon dioxide (CO_2_) and oxygen (O_2_) was determined at 873 and 1308 mL·m^−2^ day^−1^ for neat PLA, respectively, followed by 260 and 104 mL·m^−2^ day^−1^ for PLA/ZnO: Cu/Ag (0%), and 230 and 107 mL·m^−2^ day^−1^ for 0.5 wt.% of PLA/ZnO: Cu/Ag films, respectively (Table 3). At high concentrations of ZnO: Cu/Ag nanoparticles, their heterogeneous aggregation and dispersion can reduce and negatively affect these barrier properties. According to the results of this investigation, 0.5% ZnO: Cu/Ag NPs have the best barrier characteristics and are appropriate for packing.

In a study by Cardenas et al. [162], water vapor permeability (WVP), and moisture content on pure chitosan biopolymer and chitosan-CuONPs nanocomposite film were investigated. The films were exposed for two *h* at 20°C and 50% RH before the WVP examination (Table 3).

WVP is an essential factor in detecting water vapor permeability and removal from the matrix. Chitosan films showed higher WVP than films containing copper oxide nanoparticles. The WVP for chitosan and chitosan-CuONP films determined 3 × 10^−3^ and 3 × 10^−4^ (g mm/m^2^ day kPa). Copper oxide nanoparticles increased water resistance and decreased WVP resistance. This may be due to the interaction of hydrogen bonds between chitosan and solvent area or roughness affecting the permeability. Equilibrium moisture content (EMC) for chitosan-copper oxide film was lower than that for the chitosan film due to the colloidal solution used in its preparation. The addition of metal nanoparticles due to ionic or bipolar-dipole bonding to the matrix biopolymer reduces the water's impact on the film. The EMC of the chitosan film was determined 33.7%, while that of the chitosan-CuO nanoparticles film was 6.1% (Table 3).

In the study by Saravanakumar et al. [149], they produced combination films with cellulose, sodium alginate (SA), and CuO nanoparticles with various concentrations. The moisture content was different between the films of CuO nanocomposite biopolymers. The highest amount was related to sodium alginate (3%)-CuONPs (5 mM) biopolymer with about 14.7% moisture content. Then, CNW (0.5%)-SA (3%)-CuoNPs (5 mM) was about 13%. The moisture content of sodium alginate (1%)-CuONPs (1 mM) and sodium alginate (3%)-CuONP (1 mM) nanocomposite biopolymers was about 12.5% (Table 3).

In another study conducted by Hasheminya and his colleagues [163], they investigated the kefiran-CMC biopolymer's mechanical properties by increasing the concentration of copper oxide nanoparticles from 0.5%, 1%, and 2% (Table 3). As the concentration of copper oxide nanoparticles increased, water permeability decreased. So, the nanocomposite with a concentration of 0.5% had the lowest water permeability. The WVP for every biocomposite film was 2.30, 2.65, and 3.81 (10^−7^ g m/m^2^ Pa.h) for kefiran-CMC-CuONPs (2%), kefiran-CMC-CuONPs (1%), and kefiran-CMC-CuONPs (0.5%), respectively. Copper oxide nanoparticles have a low hydrophobicity and permeability compared to the polymer matrix. On the other hand, the gaps between the polymer chains were filled in, thus reducing the mobility of the chain and reducing WVP [139] (Table 3).

The WVP for every biocomposite films was 2.30, 2.65, and 3.81 (10–7 g m/m2 Pa.h) for kefiran-CMC-CuONPs (2%), kefiran-CMC-CuONPs (1%), and kefiran-CMC-CuONPs (0.5%), respectively. Copper oxide nanoparticles have a low hydrophobicity and permeability compared to the polymer matrix. On the other hand, the gaps between the polymer chains were filled, which reduces the mobility of the chain and reduces WVP [140] (Table 3).

### 3.5. Antioxidant Activities in CuO-Carbohydrate Films

Active antioxidant substances are active packaging types, especially for supplements and additives, to maintain food quality in transportation and packaging. This is due to their penetration into the enzymatic systems of some pathogens [192]. This innovative packaging is designed to maintain food quality against oxidation [193]. Aerobic bacteria and insects need oxygen to grow so that oxygen absorbers can prevent them from growing [194]. Because oxygen can easily pass through different membranes, exposure to excess oxygen can lead to microorganisms' growth [195]. Changes in antioxidants' structure can affect their shielding effect, and the food may spoil after a short time [196,197]. Oxidation of fats, pigments, and oxygen-sensitive vitamins such as vitamins A and C can alter the texture, taste, odor, and quality of food [198].

Most importantly, such chemical changes cause toxic aldehydes due to the destruction of unsaturated fatty acids [199]. Polymeric antioxidants are a group of substances that exhibit potent antioxidant and medicinal properties compared to low-molecular-weight compounds. They are made from polymerizing antioxidant molecules or biopolymer compounds (naturally and artificially) [192]. Antioxidant polymers have both polymeric and antioxidant properties. Enzymatic or chemical methods can be used to synthesize antioxidants. The obtained materials can retain their antioxidant properties and have excellent resistance in the macromolecular systems [192]. Proper selection of antioxidant compounds in food packaging is essential. Combining antioxidants and lubricants to achieve a one-handed product should be considered.

On the other hand, the type of antioxidant should be suitable for the type of food; for example, the polar antioxidant is ideal for packing high-fat foods and vice versa. This condition is called the “antioxidant paradox” [199]. Edible coating technology is an active nanopackaging that can reduce food spoilage. The primary mechanism is reducing the oxygen transfer rate and the possibility of incorporating antioxidant substances into the coating matrix or edible film. One solution is to use nanoparticles in the matrix coating [199]. The role of copper oxide nanoparticles in edible films is discussed below. Revathi et al. [56] investigated copper nanoparticles' antioxidant role in an edible biopolymer using the DPPH and ABTS method. This study analyzed the antioxidant properties of copper oxide, chitosan, chitosan-CuO, NS-CuO, and chi-CuO-NS biocomposites at different concentrations (5, 25, 50, 75, and 100 *μ*g/mL). The DPPH radical scavenging assay obtained the highest EC50 for chitosan with 92.55 *μ*g/mL, followed by CuO, chi-CuO, NS-CuO, and chi-CuO-NS with 91.71, 90.03, 88.03, and 76.02 *μ*g/mL, respectively. Based on the EC50, chi-CuO and NS-CuO have significant radical scavenging activity compared to CuO and are similar to its result. This may be due to the phytochemicals in neem and chitosan attached to the copper oxide [200] (Table 4). The CS-CuO-NS biocomposite at 100 *μ*g/mL concentration showed about 59% and 56% radical scavenging activity for DPPH and ABTS. While positive control, ascorbic acid was inhibited at the same concentration of about 70% and 72%, respectively. The ABTS radical scavenging assay for chitosan obtained the highest EC50 value with 92.06 *μ*g/mL, among other composites followed by CuO, CS-CuO, NS-CuO, and CS-CuO-NS with 91.05, 90.95, 89.99, and 88.53 *μ*g/mL, respectively (Table 4).

Besides, the percentage of inhibition in chitosan-CuO biocomposite is similar to copper oxide particles because chitosan has less antioxidant activity due to its intramolecular hydrogen bonding. In general, incorporating the pyridinium group into the biopolymer structure can destroy part of the hydrogen bonds, convert the amine group to imine, and affect the part of the antioxidant property of the chitosan related to the amine group [211]. In chitosan, there are hydroxyl and amine groups that play an essential role in its antioxidant properties. However, intramolecular hydrogen bonds in the chitosan molecules reduce these two groups [212].

In the study by Saravanakumar et al. [37], they evaluated the DPPH and ABTS scavenging activity with four produced combination films with cellulose, sodium alginate (SA), and CuO nanoparticles with various concentrations. The CNW (0.5%)-SA (3%)-CuONPs (5 mM) film showed the highest DPPH and ABTS scavenging activity with 46.55% and 35.46% compared to other films, respectively. Active food packaging liberated copper oxide nanoparticles and CNW against ABTS and DPPH oxidation. Copper oxide nanoparticles and CNW are linked to the biopolymers' antioxidant properties [200,213] (Table 4).

The DPPH scavenging activity was estimated for sodium alginate (1%)-CuONPs (1 mM), sodium alginate (3%)-CuONPs (1 mM), and sodium alginate (3%)-CuONPs (5 mM) biopolymers with approximately 25%, 33%, and 38%, respectively. For ABTS, the scavenging activity calculated around 29%, 30%, and 20% refers to sodium alginate (1%)-CuONPs (1 mM), sodium alginate (3%)-CuONPs (1 mM), and sodium alginate (3%)-CuONPs (5 mM) biopolymers, respectively (Table 4). Active food packaging released copper oxide nanoparticles and CNW against ABTS and DPPH oxidation. The antioxidant activity in the biopolymer is related to the concentration of copper oxide nanoparticles. Because of the increase in copper oxide nanoparticles' concentration, the released electrons are bound to the DPPH-free nitrogen atom [214, 215]. Overall, increasing the concentration of CuONPs increased DPPH scavenging inhibition and ABTS scavenging in this investigation.

The studies above clearly show that copper oxide nanoparticles have better antioxidant properties than edible films without nanoparticles. It can also be concluded from the above studies that as the concentration of copper oxide nanoparticles increased, its antioxidant property increased because their electrons transferred into the free site of the nitrogen atom and occupied it, and the inhibition rate increased.

### 3.6. Antimicrobial Activities in CuO-Carbohydrate Films

In food packaging, the development of microorganisms such as bacteria, fungus, yeast, and viruses is a serious problem. Using robust antimicrobial packaging can be the right solution for creating shelf life, quality, and food safety against pathogens that cause food to spoil [216, 217]. Antimicrobial packaging is an active packaging type. In addition to its other beneficial properties, active packaging can play an essential role in controlling and preventing the growth of pathogens in the food packaging [218–220]. They naturally or artificially produce antimicrobial compounds in the packaging structure [221, 222]. Producing new packaging with natural antimicrobial compounds is a promising way to protect food and is one of the factors used today with nanoparticle coatings [223, 224]. Metal oxide nanoparticles are among minerals that have natural antimicrobial activity. This polymer-assisted ensures that the nanoparticles are evenly dispersed throughout the polymer matrix, impacting the films' thermal stability, mechanical strength, barrier properties, homogeneity, and antimicrobial properties [44]. This type of antimicrobial material has higher thermal stability than organic matter, having the same property. Therefore, metallic nanoparticles (MNP) are highly resistant to harsh conditions [73, 225]. One of these metal nanoparticles is copper oxide, which has been investigated for its antimicrobial properties in various biopolymers film in different studies [128, 135, 143, 145, 163, 205, 226, 227] (Tables 5 and 6).

Various studies have shown that chitosan as an edible film is combined with copper oxide nanoparticles and exhibits excellent antimicrobial properties. The authors concluded that the composition of copper oxide nanoparticles homogeneously in the edible film could play a role in this respect. To this end, they investigated the antimicrobial role of CuO nanocomposite biopolymers such as CMC, chitosan, cellulose, BC, alginate on the microorganisms of *E. coli*, *B. subtilis*, *S. aureus*, *K. pneumonia*, *P. aeruginosa*, *P. Vulgaris*, *P. mirabilis*, and *P. chrysogenum* (*P. notatum*). Researchers examined the disc's antimicrobial properties in these studies and reported a growth inhibition rate.

Jayaramudu et al. [202] pointed to the antimicrobial role of this chitosan-CuONP biopolymer. The inhibition efficiency of the bacteria was measured after 48 h of growth at 37°C. In this study, it was found that inhibition efficiency was in *E. coli* compared to *Bacillus*. They showed the 16 mm and 13 mm inhibition zone (IZ) *for E.coli* and *Bacillus*, respectively (Table 5). This report corroborates with other reports that copper oxide nanoparticles have more potent antimicrobial properties than copper nanoparticles [228, 229]. In vitro condition, Farhoudian et al. [148] demonstrated that the antibacterial activity of chitosan-CuO was recorded on Gram-negative bacteria (*E. coli*) and Gram-positive (*S. aureus*) by the disk diffusion test. They presented the 6–11 mm and 6–8 mm inhibition zone for *E.coli* and *S. aureus*, respectively, for chitosan-CuO at different times (Table 4).

Logpriya et al. [210] presented similar results to Farhoudian et al. They examined the antimicrobial activity of chi-CuO at 25, 50, and 100 *μ*L. They showed the IZs for *E. coli* at 25 *μ*Land 50 *μ*L to be 6 mm, and at 100 *μ*L to be 8 mm. The IZs for *B. subtilis*, *P. aeruginosa*, and *P. notatum* at 25, 50, and 100 *μ*L concentrations were 6.5, 7, 8 mm; 6, 6.5, 8 mm; and 0, 10, 10 mm, respectively (Table 5). From their study, it can be concluded that antimicrobial activity increased with an increase in the concentration of chitosan-CuO biopolymer. The antimicrobial activity usually depends on the size and shape of the copper oxide nanoparticles [230]. Another study showed that copper oxide nanocomposites with biopolymer coatings had a potent antimicrobial activity [231]. Due to its many hydroxyl and amine groups, chitosan has a high affinity to metal ions, leading to the formation of chitosan-copper oxide nanocomposites [232].

In another study [56], the antimicrobial properties of chitosan, CuO, chitosan-CuO, neem seed-CuO, and chi-CuO-NS biocomposites were investigated. This examination reviewed these nanocomposites' antimicrobial properties on two significant strains of Gram-positive pathogenic bacteria, *S. aureus*, *S. pyogenes*, and two strains of Gram-negative bacteria *E. coli* and *K aerogenes* by agar well diffusion (Table 5). The chi-CuO-NS showed the highest antimicrobial activity against *S. aureus* with a 23-mm inhibition zone among these nanocomposite films, followed by *E. coli*, *S. pyogenes*, and *K. aerogenes* showing 22-, 21-, and 20-mm inhibition zones, respectively. NS-CuO's next film represented 20 mm IZ for *S. aureus*, followed by *S. pyogenes*, *K. aerogenes*, and *E. coli* with 19 mm, 19 mm, and 18 mm, respectively. Chitosan-CuO calculated a 19 mm inhibition zone for *S. aureus*, followed by *E. coli*, *S. pyogenes*, and *K. aerogenes* with 18, 18, and 17 mm inhibition zone, respectively. The CuO nanoparticle individually showed the 17 mm inhibition zone for *S. aureus*; and then, 16 mm, 16 mm, and 15 mm inhibition zone for *E. coli*, *S. pyogenes*, and *K. aerogenes*, respectively. Finally, chitosan showed moderate antimicrobial activity against *S. aureus* with a 15 mm inhibition zone, followed by 14 mm, 13 mm, and 12 mm IZ for *S. pyogenes*, *E. coli*, and *K. aerogenes*, respectively, compared to other nanocomposites. From this study, it can be concluded that chitosan, CuO, chitosan-CuO, neem seed-CuO, and chi-CuO-NS biocomposites confirmed a zone of inhibition against four pathogens in the order *S. aureus* *>* *E. coli* *>* *S. pyogenes* *>* *K. aerogenes*, respectively (Table 5). In this study, MIC and MBC tests were performed for *S. aureus*.  Table 6 shows all the nanocomposites synthesized against this bacterium. It can be deduced from this table that the CS-CuO-NS biocomposite increased the antimicrobial activity at concentrations of 25 to 100 *μ*g/mL. In addition to the previously mentioned reason [40,41], there is another possibility for bacteriolysis, which is the production of reactive oxygen species (ROS) due to metal oxide [215]. The ROS interact with the microbial cell membrane, which leads to cell lysis and bacteria death. Reactive oxygen species contact the bacterial membrane and cause lysis and bacterial death [215].

Al-Enizi AM et al. [205] showed the inhibition zone against UTI pathogens (*E.coli, S. aureus, K. pneumonia, P. aeruginosa, P. Vulgaris,* and *P. mirabilis*) (Table 5). Their study investigated antimicrobial activity for CuNPs-CMC hydrogel at three concentrations 1%, 3%, and 5%, respectively. The IZ for *E. coli* demonstrated 12, 13.6, and 15.2 mm at 1%, 3%, and 5% concentration, respectively. For *S. aureus*, IZ was 12.2, 14, and 15.6 mm at 1%, 3%, and 5% CuNPs-CMC hydrogel concentrations, respectively. For *K. pneumonia* at the same order of concentrations, IZ was 11.4, 13.2, and 15.2 mm, respectively. The inhibition zone for *P. aeruginosa* was 12.8, 14, and 16.4 mm at 1%, 3%, and 5% CuNPs-CMC hydrogel concentration, respectively. *P. Vulgaris* showed the 12 mm, 13.8 mm, and 15.8 mm inhibition at the same order of concentrations, respectively. For *P. mirabilis,* the inhibition zone was showed 12.8 mm, 14 mm, and 15.8 mm at 1%, 3%, and 5% CuNPs-CMC hydrogel concentrations, respectively (Table 5).

CMC-CuO demonstrated a comparable antibacterial activity on *E. coli* and *S. aureus* in research by Yadollahi et al. [147]. *E. coli* displayed a 9–14 mm inhibition zone at varying doses, whereas *S. aureus* showed a 15–19 mm inhibition zone (Table 5). The results show that the nanocomposite hydrogel with copper oxide was more toxic to the bacteria under the same conditions than pure hydrogel. The study also showed that nanocomposite hydrogels' antimicrobial efficiency, regarding the bacterial type, was affected by the concentration of copper oxide nanoparticles because the hydrogels with higher nanoparticles showed significant antimicrobial properties.

To investigate the antimicrobial properties of cellulose-CuNPs, Muthulakshmi and her colleagues [201] experimented on *E. coli* bacteria (Table 5). Antimicrobial activity tests were also performed on cellulose-free nanoparticles (as control). Antimicrobial results were assessed on control samples and cellulose-Cu nanocomposites for concentrations of 5 mM, 25 mM, 125 mM, and 250 mM of copper sulfate solution. Although the control sample showed no antimicrobial activity, all the copper nanoparticle biocomposites had significant antimicrobial properties. For concentrations of 5 mM, 25 mM, 125 mM, and 250 mM of copper sulfate solution, they obtained inhibitory zones of 2 mm, 5 mm, 10 mm, and 12 mm for cellulose-Cu nanocomposites (Table 5).

In another study by Muthulakshmi and her colleagues [208], they reported the potential inhibition of the cellulose-CuNP nanocomposite films at 250 *μ*g/ml and 500 *μ*g/ml of CuNP in the matrix against *Bacillus* and four concentrations against *E. coli* strains. The cellulose-CuNP (250 mM) nanobiocomposite shows the highest inhibition rate by 12 mm for *E. coli*. After that, 125 mM concentration showed the 9 mm inhibition zone. For cellulose-CuNP, 5 mM and 25 mM concentrations did not show any IZ against *E. coli* bacteria (Table 5).

Two concentrations of cellulose-CuNP, 250 mM and 500 mM, were considered for antimicrobial activity against *Bacillus* bacteria. The results demonstrated a 29 mm and a 32 mm inhibition zone for zone clearance for cellulose-CuNP (250 mM) and cellulose-CuNP (500 mM).

## 4. Conclusion

**Figure 1:**
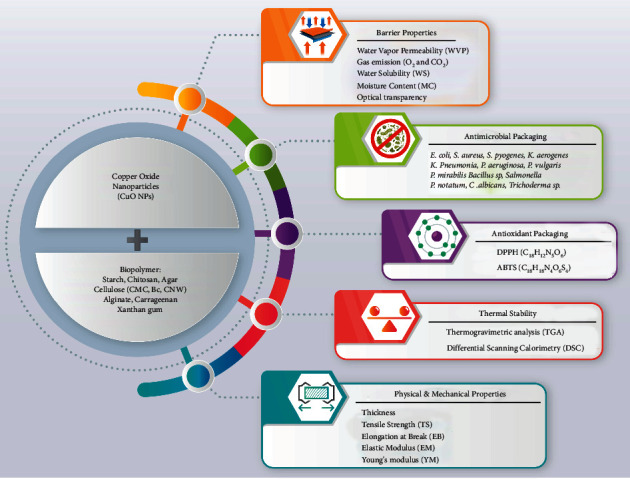
Advantageous effects of copper oxide nanoparticles on the physical, mechanical, barrier, thermal stability, antioxidant, and antimicrobial properties of carbohydrate-based films in food nanopackaging.

One of the most pressing challenges today is to remove or reduce the amount of plastic that is most damaging to the environment. Biopolymers for biodegradable and alternative plastics have been the subject of substantial research in recent years. Biopolymers can help to reduce waste by reducing the usage of synthetic and chemical compounds. However, biopolymers alone have mechanical, thermal stability, antimicrobial, and antioxidant properties. Nanotechnology nowadays comes with the help of biopolymers to enhance their physical, mechanical, and barrier properties. Copper nanoparticles and their derivatives are among the most critical nanoparticles in this study (Figure 1). The combination of copper nanoparticles with biopolymers increases their mechanical properties (such as tensile properties and flexibility) and improves their barrier properties (such as water vapor permeability, oxygen gas, and carbon dioxide emissions). Improving barrier properties can play an essential role in active food packaging. Combining copper nanoparticles with biopolymers reduces oxygen penetration in food packaging. It prevents aerobic bacteria's growth under anaerobic conditions, thus having a potent antimicrobial property that synthetic products such as plastics lack. On the other hand, reducing oxygen levels does not cause the compounds of active oxygen species to impair the food's antioxidant properties. However, further studies are needed on the role of copper nanoparticles and their derivatives with biodegradable biopolymers to find a more suitable option for active packaging without causing toxicity and endangering the environment and human health.

## Figures and Tables

**Table 1:** Biodegradable polymers used in food packaging.

Polysaccharides	Proteins	Aliphatic polyesters
Agar	Collagen	Polylactic acid (PLA)
Alginate	Gelatin	Polyhydroxy butyrate (PHB)
Carrageenan	Whey protein	
Cellulose	Soy protein	
Chitin/Chitosan	Zein	
Curdlan		
Gellan		
Pectin		
Pullulan		
Starch		
Xanthan		

**Table 2:** Physical properties of copper oxide biocomposite films as food packaging applications in different studies.

Study	Sample of films (weight %)	Thickness (*μ*m)	Tensile strength (TS) (MPa)	Elongation at break (EB) (%)	Elastic modulus (EM) (GPa)	Young's modulus (YM) (MPa)	TGA	*T *_g_ (°C)
[142]	Agar-CuONPs	60.9 ± 2.7	38.6 ± 3.0	20.3 ± 3.8	1.21 ± 0.12	Not reported	259°C	Not reported
	Alginate-CuONPs	50.7 ± 3.8	45.9 ± 6.9	18.4 ± 6.4	1.34 ± 0.37		219°C	
	Carrageenan-CuONPs	52.0 ± 2.7	55.5 ± 8.9	11.0 ± 2.7	1.62 ± 0.23		253°C	
	Chitosan-CuONPs	62.1 ± 9.6	52.9 ± 4.2	21.8 ± 5.8	1.77 ± 0.16		264°C	

[159]	Starch	120 ± 5 *μ*m	3.67 ± 0.31	98.00 ± 2.00	Not reported	7.80 ± 1.14	Not reported	Not reported
	Starch-Ag		4.17 ± 0.21	42.67 ± 2.52		14.05 ± 1.21		
	Starch-ZnO		7.50 ± 0.40	55.67 ± 2.08		25.44 ± 2.01		
	Starch-CuO		6.03 ± 0.15	65.00 ± 2.00		22.82 ± 1.78		
	Starch-Ag-ZnO-CuO		4.93 ± 0.23	76.67 ± 2.08		19.87 ± 1.34		

[160]	Neat PLA	Below 200 *μ*m	69.28 ± 0.18	2.14 ± 0.10	Not reported	4048 ± 17	Not reported	57.5
	PLA/ZnO : Cu/Ag (0%)		44.81 ± 9.19	3.30 ± 0.64		2898 ± 149		44.0
	PLA/ZnO : Cu/Ag (0.5%)		45.32 ± 7.53	2.78 ± 0.23		2934 ± 161		46.4
	PLA/ZnO : Cu/Ag (1%)		48.39 ± 5.35	2.67 ± 0.52		3058 ± 72		48.5
	PLA/ZnO : Cu/Ag (1.5%)		47.28 ± 2.75	2.61 ± 0.27		3010 ± 107		47.5

[161]	CuO-cellulose	Not reported	Not reported	Not reported	Not reported	Not reported	Not reported	70–150
	Chitosan-cellulose		30.58					
	CuO-chitosan-cellulose		59.77					
	CuONPs-chitosan-cellulose		38.88					

[162]	Chitosan film	46 ± 7	Not reported	Not reported	Not reported	Not reported	Not reported	70–140
	CuNPs-chi film	53 ± 4						

[149]	Sodium alginate (1%)-CuONPs (1 mM)	∼58	Not reported	Not reported	Not reported	Not reported	Not reported	Not reported
	Sodium alginate (3%)-CuONPs (1 mM)	∼62						
	Sodium alginate (3%)-CuONPs (5 mM)	∼67						
	CNW (0.5%)-SA (3%)-CuoNPS (5 mM)	∼71						
	Sodium alginate (1%)-CuONPs (1 mM)	∼58						

[163]	Kefiran-CMC-CuONPs (0.5%)	107 ± 6	3.62 ± 0.05	71.78 ± 1.57	Not reported	Not reported	Not reported	25.43
	Kefiran-CMC-CuONPs (1%)	110 ± 10	4.12 ± 0.05	64.41 ± 2.61				44.81
	Kefiran-CMC-CuONPs (2%)	117 ± 6	4.48 ± 0.16	60.74 ± 0.68				83.13

**Table 3:** Food barriers of copper oxide biocomposite films as food packaging applications in different studies.

Study	Film sample	Vis light (600 nm)	WVP	WS (%)	RH	CO_2_ (mL·m^−2^ day^−1^)	O_2_ (mL·m^−2^ day^−1^)	Moisture (%)
[142]	Agar/CuONPs	13.9 ± 0.7	1.18 ± 0.06	Not reported	50% RH for 48h, 25°C	Not reported	Not reported	Not reported
	Alginate/CuONPs	14.3 ± 0.4	1.35 ± 0.17					
	Carrageenan/CuONPs	15.8 ± 0.9	1.19 ± 0.08					
	Chitosan/CuONPs	75.4 ± 0.9	1.03 ± 0.17					
	CMC/CuONPs	9.63 ± 0.5	1.40 ± 0.08					

[159]	Starch	Not reported	11.46 ± 0.23	32.25 ± 0.85	50 ± 5% RH for 48h, 25°C	Not reported	Not reported	Not reported
	Starch-Ag (1%)		10.49 ± 0.14	30.13 ± 0.74				
	Starch-ZnO (1%)		10.15 ± 0.14	27.57 ± 0.81				
	Starch-CuO (1%)		10.79 ± 0.20	30.86 ± 0.60				
	Starch-Ag-ZnO-CuO		10.70 ± 0.24	30.61 ± 0.47				

[160]	Neat PLA	Not reported	15.94	Not reported	Not reported	873	1308	Not reported
	PLA/ZnO : Cu/Ag (0%)		13.70			260	104	
	PLA/ZnO : Cu/Ag (0.5%)		11.35			230	97	
	PLA/ZnO : Cu/Ag (1%)		18.72			Invalid test	Invalid test	
	PLA/ZnO : Cu/Ag (1.5%)		15.60			Invalid test	Invalid test	

[162]	Chitosan film	Not reported	3 × 10^−3^ ± 0.0001	Not reported	50% RH for 24h, 20°C	Not reported	Not reported	33.7 ± 1.7
	CuONPs-chi film		3 × 10^−4^ ± 0.0001					6.1 ± 2.2

[149]	Sodium alginate (1%)-CuONPs (1 mM)	Not reported	Not reported	Not reported	Not reported	Not reported	Not reported	∼12.6
	Sodium alginate (3%)-CuONPs (1 mM)							∼12.4
	Sodium alginate (3%)-CuONPs (5 mM)							∼14.7
	CNW (0.5%)-SA (3%)-CuoNPS (5 mM)							∼13

**Table 4:** Overview of copper oxide biocomposite films' studies published as food packaging applications.

Polymer + CuONPs	CuONPs' particle size	Polymer + CuONPs' particle size	Cytotoxicity (cell line-IC_50_)	Antimicrobial activities' tested strains	Antioxidant activity (DPPH-ABTS	Study
CMC-CuO	40–50 nm	60–75 nm	Not reported	*E. coli*-*S. aureus*	Not reported	[147]
Chitosan-CuO	Not reported	10–25 nm	Not reported	*E. coli*-*S. aureus*	Not reported	[148]
Chitosan	Not reported	Not reported	MCF-7 = 29.73 *μ*g/mL	*E. coli*, K. aerogenes-*S. aureus*-S. pyogenes	DPPH = 92.55 *μ*g/mL ABTS = 92.06 *μ*g/mL	[56]
CuO	Crystal size: 22 nm	------	MCF-7 = 28.82 *μ*g/mL		DPPH = 91.71 *μ*g/mL ABTS = 91.05 *μ*g/mL	
Chitosan-CuO	—	Crystal size: 23 nm	MCF-7 = 24.22 *μ*g/mL		DPPH = 90.03 *μ*g/mL ABTS = 90.95 *μ*g/mL	
Neem Seed-CuO	—	Crystal size: 82 nm	MCF-7 = 93.83 *μ*g/mL		DPPH = 88.03 *μ*g/mL ABTS = 89.99 *μ*g/mL	
Chitosan-copper oxide-neem seed (CS-CuO-NS)	—	Crystal size: 35 nm	MCF-7 = 16.33 *μ*g/mL		DPPH = 76.02 *μ*g/mL ABTS = 88.53 *μ*g/mL	
Chitosan-copper oxide (CS–CuO)	13–15 nm	10–30 nm	A549 = 20 ± 0.50 *μ*g/mL	Not reported	Not reported	[57]
*Terminalia catappa* leaf extract cellulose/CuO	21–40 nm	10–60 nm	Not reported	*E. coli*	Not reported	[201]
CuO-bacterial cellulose composite	Not reported	50–100 nm	Not reported	*E. coli*-*S. aureus*	Not reported	[58]
Chitosan-CuO	Not reported	7 ± 2 nm	Not reported	*E. coli*-*S. aureus*	Not reported	[202]
Starch-based polyurethane/CuO nanocomposite	Not reported	47.51 nm	Not reported	*E. coli*, *S. aureus*, *P. aeruginosa*, *E. faecalis* and C. albicans	Not reported	[203]
Carboxymethyl chitosan/CuO nanocomposites (CMCh/CuONPs)	Not reported	20–50 nm	Not reported	*E. coli-S. aureus*	Not reported	[204]
BC-CuO	Sonochemical CuO crystal size: 43.52 nm	Sonochemical BC crystal size: 1.67 nm	Not reported	*E. coli*-*S. aureus*	Not reported	[59]
	Precipitation CuO crystal size: 52.19 nm	Precipitation BC crystal size: 1.82 nm				
Cellulose gum and copper nanoparticles-based hydrogel (HCuNPs)	7–12 nm	Not reported	HeLa cells = 45 *μ*g/mL	K. pneumonia, *E. coli*, *P. aeruginosa*, *S. aureus*, P. vulgaris and *P. mirabilis*	Not reported	[205]
Chitosan-CuO	50–60 nm	58 nm	Not reported	Not reported	Not reported	[206]
Carrageenan and copper nanoparticles-based hydrogels and films	Not reported	150–200 nm	Not reported	*E. coli* - L. monocytogenes	Not reported	[172]
Chitosan-CuNPs	163 nm	100–200 nm	Not reported	*P. aphanidermatum-R. solani*	Not reported	[207]
Cellulose-CuNPs	20–80 nm	100 nm	Not reported	*E. coli*-*Bacillus*.sp	Not reported	[208]
Sodium alginate (1%)-CuONPs (1 mM)	Not reported	Not reported	Not reported	*S. aureus*, *E. coli*, *Salmonella* sp., C. albicans, Trichoderma spp.	DPPH = 25%ABTS = 29%	[149]
Sodium alginate (3%)-CuONPs (1 mM)					DPPH = 33%ABTS = 30%	
Sodium alginate (3%)-CuONPs (5 mM)					DPPH = 38%ABTS = 20%	
CNW (0.5%)-SA (3%)-CuoNPS (5 mM)					DPPH = 46.55%ABTS = 35.46%	
Agar-CuO nanoparticles (AG-CuO)	92 ± 15 nm	Not reported	Not reported	Not reported	Not reported	[209]
Chitosan-CuO	Not reported	Crystal size: 17 nm	Not reported	B. subtilis, *P. aeruginosa*, *E. coli*, P. notatum	Not reported	[210]
PLA/ZnO : Cu/Ag bionanocomposites	Not reported	50–100 nm	Not reported	*S. aureus*, *P. aeruginosa*	Not reported	[160]
Cotton cellulose/copper nanoparticles (CuNP)	28.9 nm	Not reported	Not reported	*E. coli*	Not reported	[161]
Cu nanoparticles/chitosan composite film	Not reported	10.6 ± 1 nm	Not reported	*S. aureus*-Salmonella typhimurium	Not reported	[162]
Kefiran-CMC-CuONPs (0.5%)	40 nm	Not reported	Not reported	*E. coli*-*S. aureus*	Not reported	[163]
Kefiran-CMC-CuONPs (1%)						
Kefiran-CMC-CuONPs (2%)						

**Table 5:** Inhibition zone of food-borne pathogens in CuONPs biopolymer films used for antimicrobial packaging.

Microorganisms	Sample	Inhibition zone (mm)	Study
*E. coli*	CMC-CuO	9–14	[147]
*S. aureus*		15–19	

*E. coli*	Chi-CuO	6–11	[148]
*S. aureus*		6–8	

*E. coli*	Chi-CuO-NS	22	[56]
*K. aerogenes*		23	
*S. pyogenes*		20	
*S. aureus*		21	
*E. coli*	NS-CuO	18	
*K. aerogenes*		19	
*S. pyogenes*		19	
*S. aureus*		20	
*E. coli*	Chi-CuO	18	
*K. aerogenes*		17	
*S. pyogenes*		18	
*S. aureus*		19	
*E. coli*	CuO	16	
*K. aerogenes*		15	
*S. pyogenes*		16	
*S. aureus*		17	
*E. coli*	Chitosan	13	
*K. aerogenes*		12	
*S. pyogenes*		14	
*S. aureus*		15	

*E. coli*	Cellulose-CuO	2 (5 mM)	[201]
		5 (25 mM)	
		10 (125 mM)	
		12 (250 mM)	

*E. coli*	BC-CuO	11.08 (pH = 7)	[58]
		10.01 (pH = 8)	
		8.90 (pH = 9)	
		8.67 (pH = 10)	
		7.62 (pH = 11)	
*S. aureus*		23.53 (pH = 7)	
		22.57 (pH = 8)	
		21.23 (pH = 9)	
		19.42 (pH = 10)	
		16.74 (pH = 11)	

*E. coli*	Chi-Cu	14	[202]
*Bacillus*		9	
*E. coli*	Chi-CuO	16	
*Bacillus*		13	
*E. coli*	Chi-Cu-Chi	6	
*Bacillus*		5	
*E. coli*	Chi-CuO-chi	10	
*Bacillus*		7.5	

*E. coli*	BC-CuO sonochemical	5.71 ± 0.65	[59]
	BC-CuO precipitation	6.33 ± 0.44	
*S. aureus*	BC-CuO sonochemical	9.37 ± 0.97	
	BC-CuO precipitation	5.21 ± 0.22	

*E. coli*	CuNPs-CMC hydrogel (1.0)	12	[205]
	CuNPs-CMC hydrogel (3.0)	13.6	
	CuNPs-CMC hydrogel (5.0)	15.2	
*S. aureus*	CuNPs-CMC hydrogel (1.0)	12.2	
	CuNPs-CMC hydrogel (3.0)	14	
	CuNPs-CMC hydrogel (5.0)	15.6	
*K. pneumonia*	CuNPs-CMC hydrogel (1.0)	11.4	
	CuNPs-CMC hydrogel (3.0)	13.2	
	CuNPs-CMC hydrogel (5.0)	15.2	
*P. aeruginosa*	CuNPs-CMC hydrogel (1.0)	12.8	
	CuNPs-CMC hydrogel (3.0)	14	
	CuNPs-CMC hydrogel (5.0)	16.4	
*P. vulgaris*	CuNPs-CMC hydrogel (1.0)	12	
	CuNPs-CMC hydrogel (3.0)	13.8	
	CuNPs-CMC hydrogel (5.0)	15.8	
*P. mirabilis*	CuNPs-CMC hydrogel (1.0)	12.4	
	CuNPs-CMC hydrogel (3.0)	14	
	CuNPs-CMC hydrogel (5.0)	15.8	

*E. coli*	Cellulose-CuNPs (5 mM)	0	[208]
	Cellulose-CuNPs (25 mM)	0	
	Cellulose-CuNPs (125 mM)	9	
	Cellulose-CuNPs (250 mM)	12	
*Bacillus*	Cellulose-CuNPs (250 mM)	29	
	Cellulose-CuNPs (500 mM)	32	

*E. coli*	SA (1%)-CuONPs (1 mM)	5.20 ± 0.54	[149]
	SA (3%)-CuONPs (1 mM)	5.58 ± 0.85	
	SA (3%)-CuONPs (5 mM)	8.72 ± 0.15	
	CNW (0.5%)-SA (3%)-CuONPs (5 mM)	12.12 ± 0.58	
*S. aureus*	SA (1%)-CuONPs (1 mM)	21.65 ± 0.62	
	SA (3%)-CuONPs (1 mM)	12.25 ± 0.84	
	SA (3%)-CuONPs (5 mM)	17.18 ± 0.45	
	CNW (0.5%)-SA (3%)-CuONPs (5 mM)	27.49 ± 0.91	
*Salmonella* spp.	SA (1%)-CuONPs (1 mM)	12.12 ± 0.15	
	SA (3%)-CuONPs (1 mM)	18.12 ± 0.64	
	SA (3%)-CuONPs (5 mM)	24.25 ± 0.48	
	CNW (0.5%)-SA (3%)-CuONPs (5 mM)	25.21 ± 1.05	
*C. albicans*	SA (1%)-CuONPs (1 mM)	22.23 ± 0.19	
	SA (3%)-CuONPs (1 mM)	17.25 ± 0.17	
	SA (3%)-CuONPs (5 mM)	21.26 ± 0.32	
	CNW (0.5%)-SA (3%)-CuONPs (5 mM)	23.35 ± 0.45	
*Trichoderma* spp.	SA (1%)-CuONPs (1 mM)	2.50 ± 0.68	
	SA (3%)-CuONPs (1 mM)	3.63 ± 0.62	
	SA (3%)-CuONPs (5 mM)	4.63 ± 0.53	
	CNW (0.5%)-SA (3%)-CuONPs (5 mM)	5.31 ± 1.16	

*E. coli*	Chi-CuO (25 *μ*L)	6 ± 0.5	[210]
	Chi-CuO (50 *μ*L)	6 ± 0.5	
	Chi-CuO (100 *μ*L)	8 ± 0.5	
*B. subtilis*	Chi-CuO (25 *μ*L)	6.5 ± 0.5	
	Chi-CuO (50 *μ*L)	7 ± 0.5	
	Chi-CuO (100 *μ*L)	8 ± 0.5	
*P. aeruginosa*	Chi-CuO (25 *μ*L)	6 ± 0.5	
	Chi-CuO (50 *μ*L)	6.5 ± 0.5	
	Chi-CuO (100 *μ*L)	8 ± 0.5	
*P. notatum*	Chi-CuO (25 *μ*L)	Not reported	
	Chi-CuO (50 *μ*L)	10 ± 0.5	
	Chi-CuO (100 *μ*L)	10 ± 0.5	

**Table 6:** The antibacterial activity of CuONP-biopolymer films by MBC, MIC, and CFU methods in the published studies.

Microorganisms	Sample				Study
		*MIC (concentration) (μg/mL)*		*MBC (concentration) (μg/mL)*	
*S. aureus*	Chi-CuO-NS	(25,50, 75,100) +		(25,50,75,100) +	[56]
	NS-CuO	(25) −/(50,75,100) +		(25) −/(50,75,100) +	
	Chi-CuO	(25,50) −/(75,100) +		(25,50) −/(75,100) +	
	CuO	(25,50,75) −/(100) +		(25,50,75) −/(100) +	
	Chitosan	(25,50,75,100) -		(25,50,75,100) −	

	Sample		*CFU (CFU/cm * ^*2*^ * )*		Study
*E. coli*	Neat PLA		2.8 ± 0.1·103		[160]
	PLA/ZnO : Cu/Ag (0%)		1.0 ± 0.2·103		
	PLA/ZnO : Cu/Ag (0.5%)		3.3 ± 0.1·103		
	PLA/ZnO : Cu/Ag (1%)		6.5 ± 0.1·103		
	PLA/ZnO : Cu/Ag (1.5%)		1.0 ± 0.1·104		
*S. aureus*	Neat PLA		2.3 ± 0.4·104		
	PLA/ZnO : Cu/Ag (0%)		1.9 ± 0.2·104		
	PLA/ZnO : Cu/Ag (0.5%)		4.0 ± 0.1·103		
	PLA/ZnO : Cu/Ag (1%)		6.3 ± 0.1·102		
	PLA/ZnO : Cu/Ag (1.5%)		2.05 ± 0.2·102		

	Sample		*Concentration (cells/ml)*		Study
*S. aureus*	Chitosan		3 × 10^5^		[162]
	Chitosan-CuNPs		1 × 10^2^		
*S. typhimurium*	Chitosan		1.8 × 10^5^		
	Chitosan-CuNPs		2.1 × 10^3^		

## Data Availability

There are no primary raw data associated with this review.
